# MCPIP1/Regnase-1 Expression in Keratinocytes of Patients with Hidradenitis Suppurativa: Preliminary Results

**DOI:** 10.3390/ijms22147241

**Published:** 2021-07-06

**Authors:** Piotr K. Krajewski, Weronika Szukała, Agata Lichawska-Cieślar, Łukasz Matusiak, Jolanta Jura, Jacek C. Szepietowski

**Affiliations:** 1 Department of Dermatology, Venereology and Allergology, Wroclaw Medical University, Chalubinskiego 1, 50-368 Wroclaw, Poland; piotr.krajewski@student.umed.wroc.pl (P.K.K.); luke71@interia.pl (Ł.M.); 2 Department of General Biochemistry, Faculty of Biochemistry, Biophysics and Biotechnology, Jagiellonian University, Gronostajowa 7, 30-392 Krakow, Poland; weronika.szukala@doctoral.uj.edu.pl (W.S.); jolanta.jura@uj.edu.pl (J.J.)

**Keywords:** MCPIP1, hidradenitis suppurativa, Regnase-1

## Abstract

The pathogenesis of hidradenitis suppurativa (HS) is yet to be fully understood. However, inflammation is a key element in the development of skin lesions. The aim of this study was to evaluate the expression of monocyte chemotactic protein-1-induced protein-1 (MCPIP1) in the skin of patients suffering from HS. Skin biopsies of 15 patients with HS and 15 healthy controls were obtained and processed for immunohistochemistry, western blot, and real time PCR. The highest mean MCPIP1 mRNA expression was found in the inflammatory lesional skin of HS patients. It was significantly higher than MCPIP1 mRNA expression in the biopsies from both healthy controls and non-lesional skin of HS patients. Western blot analysis indicated that expression of MCPIP1 was elevated within both lesional and non-lesional skin compared to the healthy control. The increased MCPIP1 mRNA and protein expression level in HS lesions may indicate its possible role in the disease pathogenesis.

## 1. Introduction

Inflammation is a basic immune response of our organism that enables survival during infections or injuries. On the molecular level, it is a set of interactions between inflammatory factors and cells, often described as a stress response of tissue or organism to noxious conditions [1,2]. Although usually beneficial and life-preserving, disturbances of the innate immune system may lead to development of immune-mediated diseases [3]. Moreover, failure in neutralizing acute response often causes chronic inflammation and severe metabolic consequences [4]. Many of the important immune responses are carried out in the skin [5].

Hidradenitis suppurativa (HS) is a debilitating skin disorder of a complex pathogenesis, which remains unclear [6]. The role of overproduction of inflammatory cytokines and an inability of its inhibition has been mentioned by many authors [7]. The possible suppression of inflammation is of benefit for the treatment of HS [8,9,10].

Monocyte chemotactic protein-1-induced protein-1 (MCPIP1), also known as Regnase-1, is an RNase protein encoded by the ZC3H12A gene. It regulates the inflammatory activation and maintains immune homeostasis by selectively promoting the destabilization of mRNAs of certain proinflammatory cytokines (e.g., IL-6 and IL-1β) and transcription factors [11,12,13,14,15]. The lack of MCPIP1 in mice resulted in systemic inflammation leading to growth retardation, anemia, splenomegaly, lymphadenopathy, and premature death [11,16,17,18]. Recent studies indicated that in the skin, MCPIP1 functions as an important regulator of epidermal homeostasis. The ZC3H12A gene is induced by many inflammatory mediators including IL-17 and IL-36 [19,20,21]. Our previous studies on the influence of MCPIP1 on keratinocytes showed that its deficiency leads to the skin barrier impairment and spontaneous cutaneous inflammation [22]. On the other hand, MCPIP1 was shown to be upregulated in human psoriatic skin [20,23], whereas its deficiency in mice led to the much aggravated psoriasis-like inflammation phenotype induced by imiquimod [21,23,24].

We hypothesized that MCPIP1 may be involved in the pathogenesis of other than psoriasis skin disorders of inflammatory background, like HS. The aim of this study was to evaluate the expression of MCPIP1, both on mRNA and protein level, in the skin of patients suffering from HS. To the best of our knowledge, this is the first study to investigate the possible association between MCPIP1 and the pathogenesis of HS.

## 2. Results

### MCPIP1 Is Aberrantly Expressed in Hidradenitis Suppurativa

**Figure 1:**
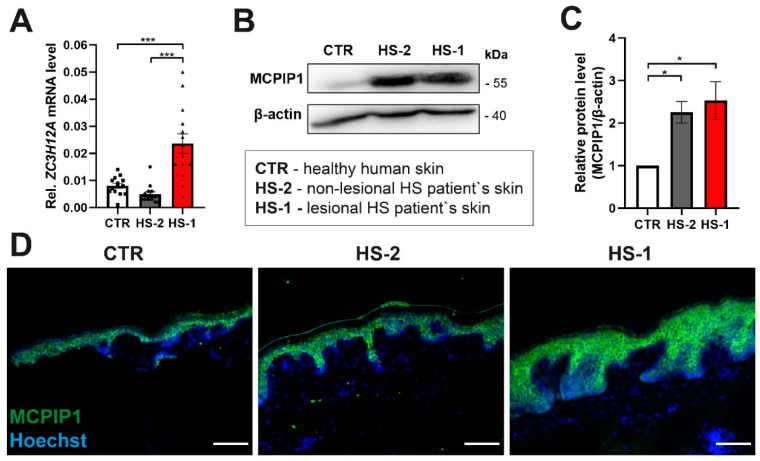
Increased expression of monocyte chemotactic protein-1-induced protein-1 (MCPIP1) in the hidradenitis suppurativa skin. (**A**) qRT-PCR analysis of MCPIP1 (ZC3H12A) transcript level in the healthy human skin (CTR), non-lesional hidradenitis suppurativa patients’ skin (HS-2) and lesional HS patients’ skin (HS-1) (*n* = 14). (**B**) Representative Western blot for MCPIP1. β-actin was used as the loading control. (**C**) Densitometric quantification of MCPIP1 protein level (*n* = 4). (**D**) Representative MCPIP1 immunofluorescence staining of the skin sections. Scale bar 100 μm. Data represent the mean ± SEM. * *p* < 0.05, *** *p* < 0.001 by one-way ANOVA.

To investigate potential association of MCPIP1 with HS, we analyzed the expression of MCPIP1 on both mRNA and protein levels in the lesional and non-lesional skin of HS patients, and healthy controls. The highest mean MCPIP1 mRNA expression was found in the inflammatory lesional skin of HS patients (HS-1: lesional skin) (0.0236 ± 0.0134). It was significantly higher than MCPIP1 mRNA expression in the biopsies from both healthy controls (CTR) (0.0080 ± 0.0034, *p* < 0.001) and non-lesional skin of HS patients (HS-2: non-lesional skin) (0.0049 ± 0.0034, *p* < 0.001) (Figure 1A). There were no statistical correlations between MCPIP1 mRNA expression in lesional HS skin and well-known HS predisposing factors (obesity and smoking), as well as between sexes, those with and without family history of HS or those who had or had not suffered from juvenile acne in their adolescence (detailed data not shown). We next determined MCPIP1 protein expression in the lysates of control and HS skin. Western blot analysis indicated that expression of MCPIP1 was elevated within both lesional (2,5-fold increase) and non-lesional skin (2,3-fold increase) compared to the healthy control (Figure 1B,C). Subsequently, we determined the in situ expression of MCPIP1 in the skin. Generally, all biopsies showed a similar pattern of immunostaining. Specific MCPIP1 immunostaining was cytoplasmic and present in the epidermis, as well as in hair follicles. MCPIP1 immunoreactivity was found in all studied biopsies (HS-1: lesional skin, HS-2: non-lesional skin and healthy control skin) in the suprabasal layers of the epidermis. The basal layer of the epidermis showed no MCPIP1 immunoreactivity. There was also no MCPIP1 immunoreactivity in the dermis. Both lesional and non-lesional HS skin showed aberrant distribution of MCPIP1 within epidermis (Figure 1D).

**Figure 2:**
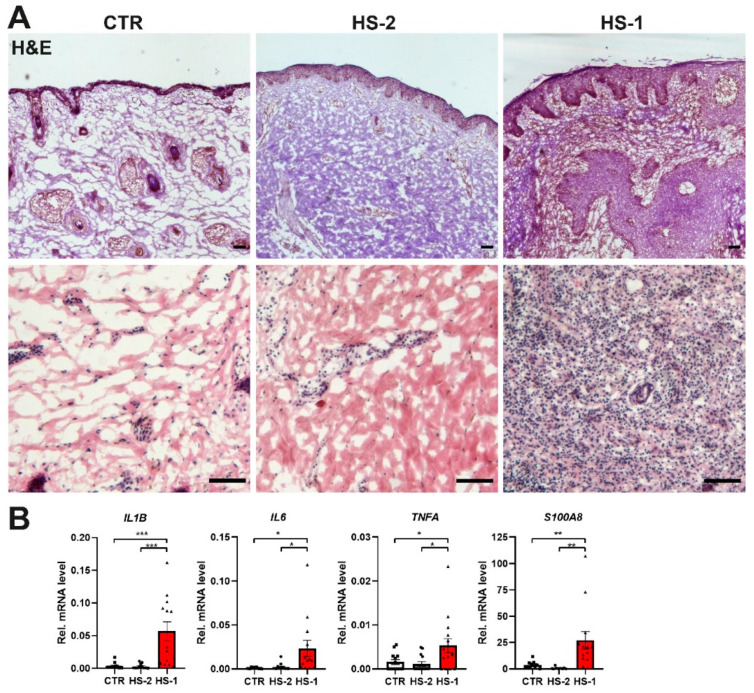
Abundant inflammation in the hidradenitis suppurativa skin. (**A**) H&E staining of the CTR, HS-1, and HS-2 skin sections at different magnification. (**B**) qRT-PCR analysis of IL1B, IL6, TNFA and S100A8 transcript level. Scale bar 100 μm. Data represent the mean ± SEM. * *p* < 0.05, ** *p* < 0.01, *** *p* < 0.001 by one-way ANOVA.

In parallel to the analyses of MCPIP1 expression, we investigated the level of inflammatory influx within HS skin. Haematoxylin and eosin staining showed a large inflammatory cell infiltration into the dermis of HS lesional skin (Figure 2A). This correlated with increased gene expression level of selected inflammatory mediators: IL-1β, IL-6, TNFα and S100A8 (Figure 2B). The non-lesional HS skin did not show any signs of inflammatory reaction (Figure 2A,B).

## 3. Discussion

The pathogenesis of HS is yet to be fully understood. Among several proposed pathogenetic factors (obesity, smoking, hormonal disbalance, genetic predisposition) immunological disturbances are considered crucial for the development of HS lesions. The involvement of the immune system and disruption in the immune response have been confirmed with the favorable outcome of anti-inflammatory biologic treatment [8,9,10].

The results of our study clearly demonstrate the increased MCPIP1 mRNA and protein expression in the lesional skin of HS patients. Our results are comparable with those reported in psoriatic plaques [20,23]. Similarly to our study, MCPIP1 mRNA and protein expression was significantly increased in psoriatic skin than in healthy control skin samples. The similarities between both studies confirm a possible mutual immune-pathogenetic pathway of HS and psoriasis. Higher MCPIP1 expression and its function in inflammatory regulation may play an important role in the pathogenesis of both disorders. As hypothesized by Monin et al. [23] it is likely that the increased MCPIP1 mRNA expression reflects the ongoing inflammatory milieu, and particularly the high IL-17A levels, demonstrated in lesional skin of both HS and psoriasis. This is not surprising as the resemblances between HS and psoriasis have already been found in pathogenesis and treatment in both disorders [25].

We observed that the level of MCPIP1 protein was elevated not only within lesional skin, but also in the skin surrounding the HS lesion. In that region MCPIP1 was stabilized, most likely as a result of a highly inflammatory environment. This, however, did not correlate with increased transcriptional expression of ZC3H12A. This may be explained by the fact that MCPIP1 RNase regulates, among many other mRNAs, also its own transcript half-live [26].

To the best of our knowledge, this is the third study in which MCPIP1 expression was assessed in human skin. Ruiz-Romeu et al. [20] in healthy skin found the expression of MCPIP1 exclusively present in the granular layer of the epidermis. A similar pattern of MCPIP1 immunoreactivity was found in atopic dermatitis [20]. In contrast, Monin L et al. [23] demonstrated MCPIP1 expression distributed in the whole epidermis of the healthy skin. Our study clearly showed MCPIP1 immunoreactivity in the suprabasal layers of the epidermis of healthy controls. In HS lesional skin we demonstrated abundant MCPIP1 immunoreactivity in the suprabasal layers of the epidermis with comparable immunostaining pattern as in non-lesional HS skin and healthy control skin. In lesional psoriatic skin MCPIP1 immunoreactivity was also predominantly found in the epidermis, distributed equally in the entire epidermis [23] or in its upper layers [20]. In both studies MCPIP1 immunoreactivity was similarly localized both in lesional and non-lesional psoriatic skin [20,23].

In the lesional skin of HS, MCPIP1 is elevated on both transcriptional and translational level and it is not sufficient to resolve inflammatory processes. We noticed high inflammatory influx and elevated transcriptional expression of selected HS-related factors: IL-1β, IL-6, TNFα and S100A8. Enhanced expression of IL-6 and IL-1β was demonstrated in the lesional skin of HS patients [27]. Expression of S100A8/A9 was also shown to be elevated in HS [28]. Moreover, increased levels of TNFα in HS patient serum and skin have been reported [29,30,31,32]. 

Another molecule that may be important in HS pathogenesis is the seventh subunit of P2X receptor (P2 × 7R), which is plasma membrane channel gated by adenosine triphosphate (ATP) [33]. It is widely distributed, especially in immune system cells. Its role is to activate the NLRP3 inflammasome and promote IL-1β maturation and release. The receptor have been previously described in psoriasis, rosacea, and HS [34,35,36]. Manfredini et al. [35] found that P2X7R protein level is higher in keratinocytes, lymphocytes, and monocytes of HS skin in comparison to healthy controls [35]. Moreover, authors presented, that P2X7R has significant, yet weak association with NLRP3 inflammasome [35]. 

We are aware of limitations of our study. Firstly, our group consisted only of 15 people suffering from HS. Although the population of well diagnosed HS patients is small, the number of patients in future studies should be bigger in order to provide more accurate data. Moreover, we have focused on MCPIP1 and its expression in the skin of HS patient. Future studies aiming for correlation between MCPIP1 and well-known proinflammatory molecules, including newly described P2X7R, would play an important role in discovering the pathogenesis of HS.

## 4. Materials and Methods

The study was approved by the local Bioethics Committee of Wroclaw Medical University. The studied group included 15 patients with HS: 7 females (46.67%) and 8 males (53.55%). The mean age of the group was 35.8 ± 11.2 years. According to the mean BMI (30.1 ± 6.31 kg/m^2^) the population was considered obese. The majority (8 people, 53.6%) of the subjects were active smokers with the mean of 9.6 ± 6.1 pack-years. 7 people (46.7%) reported to suffer from juvenile acne during adolescence, while only 2 (13.3%) had a positive family history of HS. A total 86.7% (13) of patients were treated previously, with unsatisfactory results. All included patients have not been treated for HS for at least of two months before the enrollment to the study. All the patients were examined by the dermatologists experienced with HS, in order to properly assess HS severity. According to Hurley staging [37] the severity of the disease in the majority of the patients was assessed as Hurley II (8 patients, 53.3%), in 4 subjects (26.7%) as Hurley III and in the rest (3 people, 20%) as Hurley I. As for IHS4 assessment [38], on average the patients scored 15.9 ± 8.9 points, indicating severe disease. Among the HS–associated subjective symptoms, assessed with 11-point Numeral Rating Scale (NRS), pain was the most severe (4.4 ± 2.9 points), then purulent discharge (4.2 ± 2.9 points), foul smell (3.4 ± 3.4 points) and pruritus (2.5 ± 2.6 points). 9 patients (60%) had multiple body areas affected by HS, while 6 (40%) presented HS limited to one area. Among the most frequently affected areas were armpits (11 patients, 73.3%), while buttocks affectation was present in only 1 subject (6.7%).

Additionally, 15 control healthy skin samples were collected from the age and sex-matched patients who underwent surgical procedures for non-malignant skin lesions or blepharoplasty.

### 4.1. Biopsy

Prior to the biopsy, the patients got locally injected the mixture of anesthetic (2% lidocaine) and adrenaline to diminish pain and impede bleeding. Two 5-mm punch biopsies were obtained from every HS patient. One of the biopsies was taken from the active, inflammatory lesion, while the other from the healthily looking skin in close proximity from the first one (at least 2 cm).

### 4.2. RNA Isolation and Quantitative Real-Time PCR

All collected skin samples were frozen in RNAlater (Sigma-Aldrich, Saint Louis, MO, USA) and stored at −80 °C. Total RNA was extracted from tissues by homogenization in Fenozol (A&A Biotechnology, Gdynia, Poland) using a tissue homogenizer (Miccra D-1, Miccra GmbH, Germany). The purity and concentration of total RNA were assessed using a NanoDrop 1000 spectrophotometer (Thermo Fisher Scientific, Waltham, MA, USA). Subsequently, 1 μg of total RNA was reverse-transcribed with oligo(dT) primer and M-MLV reverse transcriptase (Promega, Madison, WI, USA). The cDNA was diluted 5 times, and real-time PCR was performed using a QuantStudio 3 system (Applied Biosystems, Thermo Fisher Scientific, Waltham, MA, USA) with SYBR Green qPCR master mix (A&A Biotechnology, Gdynia, Poland). The mRNA level of MCPIP1 transcript was determined relative to elongation factor-2 (EF2) by the 2^−ΔCt^ method. The following gene-specific primer pairs were used: for ZC3H12A: GGAAGCAGCCGTGTCCCTATG and TCCAGGCTGCACTGCTCACTC, for EF2: GACATCACCAAGGGTGTGCAG and TCAGCACACTGGCATAGAGGC, for IL1B: GATGTCTGGTCCATATGAACTG and TTGGGATCTACACTCTCCAGC, for IL6: GTGAAAGCAGCAAAGAGGCA and TCACCAGGCAAGTCTCCTCA, for TNFA: TAGCCCATGTTGTAGCAAACC and TGATGGCAGAGAGGAGGTTG, for S100A8: GAATTTCCATGCCGTCTACAGG and GCCACGCCCATCTTTATCACCAG.

### 4.3. Western Blot Analysis

The protein lysate from skin tissues was isolated in RIPA (Radioimmunoprecipitation assay buffer) solution with protease and phosphatase inhibitors (Roche, Basely, Switzerland) using a tissue homogenizer and then centrifuged for 20 min, 4 °C, 14,000× *g*. The protein concentrations in the tissues lysates were measured with the bicinchoninic acid assay. The electrophoresis separation was carried out in 10% polyacrylamide gel and electrotransferred to PVDF (Polyvinylidene fluoride) membranes (Merck-Millipore, Burlington, MA, USA). After the transfer, membranes were blocked for 1 h in 3% milk in Tris-buffered saline with 0.05% Tween (BioShop, Burlington, ON, Canada) followed by an overnight incubation in the primary antibody at 4 °C. On the following day, the membranes were rinsed and incubated for 1 h with the secondary antibody. The Immobilon TM Western Chemiluminescent HRP Substrate (Merck-Millipore, Burlington, MA, USA) and the ChemiDoc system (Bio-Rad, Hercules, CA, USA) were used for signal detection. Densitometric quantification was performed using ImageLab (Bio-Rad, Hercules, CA, USA). The MCPIP1 protein level was normalized to β-actin level. The following antibodies were used: rabbit anti-MCPIP1 (GTX110807; 1:2000; GeneTex, Inc., Irvine, CA, USA), mouse anti-β-actin (A1978; 1:2000; Sigma-Aldrich), peroxidase-conjugated anti-rabbit (A0545; 1:20,000; Sigma-Aldrich, St. Luis, MO, USA) and peroxidase-conjugated anti-mouse (1:20,000; BD Pharmingen).

### 4.4. Immunofluorescence Staining

Skin tissues were embedded in Tissue-Tek O.C.T. Compound (Scigen Scientific Gardena, Gardena, CA, USA) and stored at −80 °C. Then, 8 μm cryosections were cut and stained with hematoxylin and eosin (H&E) using standard protocol. For immunofluorescence staining, antigen retrieval was performed in 10 mM citrate buffer (pH 6.0) for 30 min at 95 °C. Subsequently, skin samples were blocked with 5% horse serum, 1% BSA and 0.05% Tween in PBS for 1 h and incubated with primary rabbit antibodies against MCPIP1 (1:100; GeneTex) overnight at 4 °C in blocking buffer. Bound primary antibodies were detected by incubation with secondary goat antibodies Alexa Fluor 488 anti-rabbit (A11008; 1:600; Invitrogen, Darmstadt, Germany) for 1 h at room temperature, followed by counterstaining with Hoechst 33,258 (1:2500; Thermo Scientific). After incubation, the sections were mounted in fluorescent mounting medium (Dako) and visualized in Leica DMC5400B microscope (Leica Microsystems, Wetzlar, Germany). All figures were prepared using ImageJ (National Institutes of Health, Bethesda, MD, USA).

### 4.5. Statistical Analysis

The statistical analysis of the obtained results was performed with the use of IBM SPSS Statistics v. 26 (SPSS INC., Chicago, IL, USA) software. All data were assessed for parametric or non-parametric distribution. The minimum, maximum, mean and standard deviation numbers were calculated. Analyzed quantitative variables were evaluated using Mann–Whitney U test, Spearman and Pearson correlations, while for qualitative data test Chi2 was used. One-way ANOVA was used for the comparison of mRNA and protein expression levels between two HS samples and healthy skin. A 2-sided *p* value of ≤0.05 was considered to be statistically significant.

## 5. Conclusions

As far as we know, this is the first study assessing the expression of MCPIP1 in the skin of HS patients. Our preliminary results may be of benefit for the deeper understanding of the possible immunopathogenesis of this chronic and recurrent inflammatory dermatosis. Nevertheless, though our study sheds light on possible involvement of MCPIP1 in HS pathogenesis, further studies are necessary to clarify the exact role of MCPIP1 in the pathogenesis of HS.

## Data Availability

The data obtained in this study may be available from the corresponding authors upon a reasonable request.
